# Pyruvate Kinase M2 Promotes Prostate Cancer Metastasis Through Regulating ERK1/2-COX-2 Signaling

**DOI:** 10.3389/fonc.2020.544288

**Published:** 2020-09-29

**Authors:** Wenjing Guo, Zhishuai Zhang, Guihuan Li, Xiaoju Lai, Ruonan Gu, Wanfu Xu, Hua Chen, Zhe Xing, Liping Chen, Jiabi Qian, Shiyuan Xu, Fangyin Zeng, Fan Deng

**Affiliations:** ^1^Department of Cell Biology, School of Basic Medical Sciences, Southern Medical University, Guangzhou, China; ^2^Department of Anesthesiology, Zhujiang Hospital, Southern Medical University, Guangzhou, China; ^3^Department of Clinical Laboratory, The Fifth Affiliated Hospital, Southern Medical University, Guangzhou, China

**Keywords:** pyruvate kinase M2, ERK1/2, cyclooxygenase 2, tumor metastasis, prostate cancer

## Abstract

Pyruvate kinase M2 (PKM2) is a key enzyme of glycolysis, which is highly expressed in many tumor cells, and has emerged as an important player in tumor progression and metastasis. However, the functional roles of PKM2 in tumor metastasis remain elusive. Here we showed that PKM2 promoted prostate cancer metastasis via extracellular-regulated protein kinase (ERK)–cyclooxygenase (COX-2) signaling. Based on public databases, we found that PKM2 expression was upregulated in prostate cancer and positively associated with tumor metastasis. Further analysis showed that PKM2 promoted prostate cancer cell migration/invasion and epithelial–mesenchymal transition (EMT) through upregulation of COX-2. Mechanistically, PKM2 interacted with ERK1/2 and regulated its phosphorylation, leading to phosphorylation of transcription factor c-Jun, downstream of ERK1/2, to activate COX-2 transcription by IP and ChIP assay, while inhibition of COX-2 significantly reversed the promotion effect of PKM2 on tumor metastasis *in vivo*. Taken together, our results suggest that a novel of PKM2–ERK1/2–c-Jun–COX-2 axis is a potential target in controlling prostate cancer metastasis.

## Introduction

Prostate cancer (PCa) is one of the most commonly diagnosed cancers and the third leading cause of cancer death in men around the world, particularly in developed countries (1, 2). Although the development of effective therapy for PCa metastasis had contributed to improve the survival, the metastatic disease is still incurable and lethal (3). Thus, identification of the novel potential molecular changes that lead to formation of distant metastasis is critical for the improvement of therapeutic interventions for metastatic prostate cancer, which may favor developing therapy strategies to prevent primary prostate cancer from metastasis.

Cancer metastasis is a complex series of events, including migration of cancer cells from their primary tumor, survival in circulation, attachment to the metastatic site, and proliferation as micrometastases (4, 5). During the metastasis, cyclooxygenase-2 (COX-2), a key enzyme in the conversion of arachidonic acid to prostaglandins and other eicosanoids, played an important role in promoting a more metastatic phenotype in colorectal tumor cells, while COX-2 inhibition *in vivo* can attenuate the metastatic potential of colorectal tumors in both humans and mice (6, 7). Although COX-2 is also highly expressed and correlated with angiogenesis, the contribution of COX-2 in the regulation of prostate cancer metastasis remains unclear (8).

Pyruvate kinase isoform M2 (PKM2), the key rate-limiting enzyme catalyzing the final step of glycolysis, plays a critical role in tumor glucose metabolism and is highly expressed in various types of human cancers (9, 10). PKM2 has been proposed to play a vital role in maintaining the metabolism program (11, 12), promoting proliferation (11), resistance to genotoxic treatments (13), and metastasis (14) and inducing cancer stem-like cell properties (15) in many types of cancer. Besides its roles in regulating the cancer cell, PKM2 has been proven to contribute to tumor microenvironment, such as reducing lactate production, increasing OXPHOS (9, 16). In addition, Buschow et al. provided extracellular PKM2, which has biological function and play a role in cell–cell crosstalk (17, 18). However, the specific mechanism through which PKM2 contributes to prostate cancer progression and metastasis is yet to be fully explored.

In the present study, we further addressed the high expression of PKM2 in prostate cancer, contributing to COX-2-mediated epithelial–mesenchymal transition (EMT) and tumor metastasis by ERK1/2 signaling *in vivo* and *in vitro*. Overexpression of PKM2 in DU145 and PC-3 cells significantly increased tumor invasion and migration by Transwell assay, which is attributed to COX-2 expression, which was increased in response to PKM2, while inhibition of COX-2 significantly reversed the above phenomenon. Further analysis showed that the endogenous PKM2 interacted with ERK1/2, leading to phosphorylation and nuclear location of C-JUN, subsequently binding to COX2 promoter and transcription. In summary, we supplied a novel PKM2–ERK1/2–COX-2 signaling in mediating EMT and tumor metastasis.

## Materials and Methods

### Cell Culture and Reagents

PC-3 and DU145 were purchased from the Cell Bank of the Chinese Academy of Sciences (Shanghai, China). PC-3 was cultured using DMEM/F12 (Corning, NY, United States) containing 10% fetal bovine serum (Gibco, CA, United States), and DU145 was cultured in DMEM (Corning, NY, United States) supplemented with 10% FBS. Celecoxib was obtained from MedChem Express (NJ, United States). PD98059 and U0126 were purchased form Beyotime Institute of Biotechnology (Jiangsu, China). PGE2 was from Santa Cruz (sc-201225A). Authentications of PC-3 and DU145 cells were performed by STR profiles with ABI3500xl Genetic Analyzer, and all the cells were verified to have no contamination with mycoplasma before the experiments.

### RNA Interference and Plasmids Transfection

siRNA targeting PKM2 (si-PKM2) and negative control siRNAs (si-CTL) were purchased from GenePharma (Shanghai, China). siRNA targeting COX-2 (si-COX-2) and control siRNAs were purchased from RIBOBIO (Canton, China). Plasmids were a gift by Dr. X He (19). siRNAs and plasmids were transfected by Lipofectamine 3000 (Invitrogen, CA, United States) or with Hilymax (Dojindo, Japan) following the manufacturer’s instructions.

### Generation of Stable Cell Lines

Human PKM2 lentivirus (OE-PKM2) and the negative control (Control) were purchased from OOBIO (Shanghai, China), and shRNA of the lentivirus-targeting PKM2 (LvshPKM2) and scrambled shRNA lentivirus (Mock) were obtained from GenePharma. After an infection indicated a lentivirus, puromycin was used to select stable cell lines.

### RNA Extraction and qRT-PCR

Total RNA was isolated from different prostate cancer cell lines using TRIzol reagent (Invitrogen, 15596018) according to the manufacturer’s protocol. Equal amounts of RNA were reverse transcribed into cDNA using the All-in-One First-Strand cDNA Synthesis kit. The mRNA expression in cells was measured by qRT-PCR using an ABI Step OnePlus instrument (Foster City, CA, United States). Primers for qRT-PCR were listed as follows, *pkm2*, forward 5′-CTGTGGACTTGCCTGCTGTG-3′, reverse 5′- TGCCTTGCGGATGAATGACG-3′; *cox-2*, forward 5′-GCAATAACGTGAAGGGCTGT-3′, reverse 5′-CGGGAAGA ACTTGCATTGAT-3′.

### Immunoprecipitation (IP) and Immunoblotting

Proteins from cells were incubated with antibody and precipitated with protein A/G-agarose beads. The immunoprecipitated proteins were subjected to SDS-PAGE. The immunoblotting assay was performed using the following antibodies: anti-N-cadherin (66219-1-Ig; Proteintech, Wuhan, China), anti-E-cadherin (60335-1-Ig; Proteintech), anti-MMP9 (10375-2-AP; Proteintech), anti-MMP2 (66366-1-Ig; Proteintech), anti-vimentin (60330-1-Ig; Protein tech), anti-PKM2 (A0540; ABclonal, Wuhan, China), anti-COX-2 (12282; CST, MA, United States), anti-ERK1/2 (sc-292838; Santa Cruz, CA, United States), anti-p-ERK1/2 (AP0472; ABclonal), anti-β-actin (RM2001; Ray, Beijing, China), and anti-α-tubulin (RM2007; Ray).

### Immunofluorescence Microscopy

Immunofluorescence was performed as described in the study. PKM2 and ERK1/2 were performed as described previously (20). The samples were photographed and analyzed by Zeiss LSM880 microscopy (Zeiss, Oberkochen, Germany).

### Transwell Migration and Invasion Assays

Cells (5 × 10^4^) with 1% FBS medium were seeded in the upper chamber of each Transwell (3422; Corning). Transwell migration and invasion assays were performed as described previously (20).

### Chromatin Immunoprecipitation Assay

ChIP was carried out using a kit following the manufacturer’s protocol (17–295; Millipore, MA, United States). Antibodies for ChIP were normal rabbit IgG (2729; CST) and anti-c-Jun (9165; CST). The precipitated chromatin DNA was analyzed by real-time PCR with SYBR green on an ABI Step OnePlus instrument with gene-specific primer sets. COX-2: 5′-GTGGGAGAGATTTAATGGTC-3′ and reverse 5′-GTTACCAGATGTGTTCACCAC-3′.

### Gelatin Zymography Assay

Gelatin zymography assay was measured as described previously (20). The analysis system assessed the read strip area, width, and gray value for statistical analysis.

### *In vivo* Metastasis Assays

All mouse experiments were conducted in compliance with the ARRIVE guidelines and associated recommendations (21, 22) and approved by the Institutional Animal Care and Use Committee of Southern Medical University, China. Six-week-old male BCLB/C nude mice were purchased from the Laboratory Animal Center of Southern Medical University. The total number of mice (18) was randomly divided into two sets of 6 each. The stable cells of GFP-tagged PC3 (1 × 10^6^ cells) in a volume of 100 μl were injected into the tail vein of each mouse. After 7 days, the mice were treated with celecoxib (50 mg/kg) or equal volume of vehicle once every 4 days by oral gavage for 21 days. The mice were anesthetized with 2.5% isoflurane, and the *in vivo* fluorescence distribution was measured with a mouse *in vivo* imager.

### Hematoxylin Eosin and Immunohistochemistry Stains

Hematoxylin eosin (H&E) and immunohistochemistry (IHC) were performed as described previously (23).

### ELISA

The level of PGE2 in supernatants or serum of the mice was measured by ELISA technique (KGE004B; R&D Systems, MN, United States), following the manufacturer’s instructions.

### Database Information

Data can be found at www.oncomine.org and TCGA database^1^.

### Statistical Analysis

Data in the text are expressed as mean ± standard deviation (SD) of at least three independent replicates. Data were analyzed with Student’s *t*-test, one-way ANOVA or two-way ANOVA (as appropriate) followed by LSD *post hoc* analyses using SPSS 20.0 software (IBM Corporation, Armonk, NY, United States). The value of *p* < 0.05 was considered statistically significant.

## Results

### Clinical Correlation of PKM2 Expression in Metastatic Prostate Cancer and Prognosis

**FIGURE 1:**
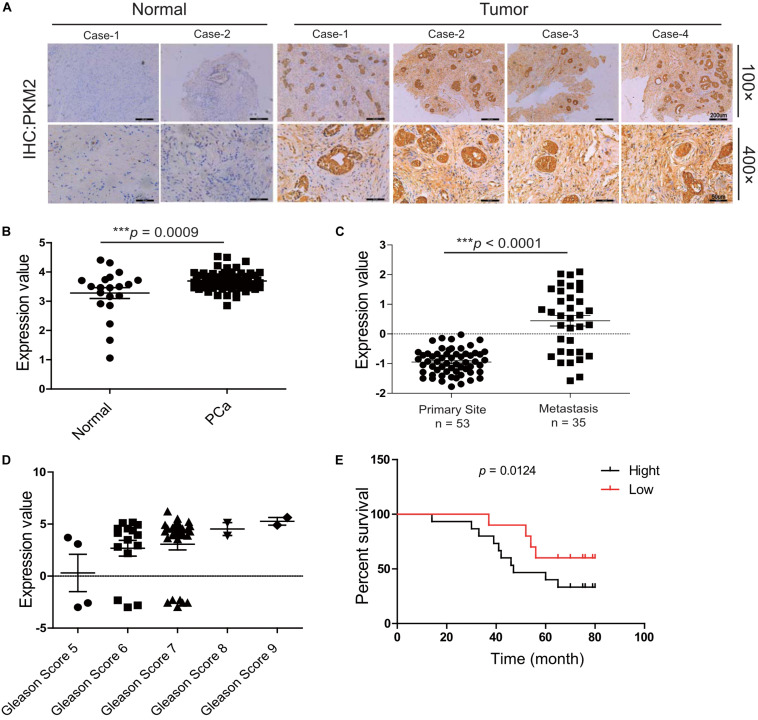
Pyruvate kinase M2 (PKM2) is overexpressed in malignant prostate tissue and predicts the poor prognosis of prostate cancer. **(A)** Expression patterns of PKM2 immunoreactivity in human prostate cancer and adjacent normal tissue; representative image of histopathologic sections of human prostate cancer and adjacent normal tissue are shown (×100); amplified images are also shown (×400). pkm2 gene expression in prostate cancer from the ONCOMINE database. **(B)** Malignant cancer tissues (*n* = 69) have a higher expression level compared with the normal tissue (*n* = 20). **(C)** Grouped by cancer sample site, pkm2 gene expression was significantly higher in metastasis (*n* = 35) than primary site (*n* = 59) (ONCOMINE database). **(D)** mRNA level of pkm2 was related to higher Gleason scores of advanced prostate cancer. **(E)** Kaplan–Meier curves demonstrate that high PKM2 expression (black, *n* = 16) in the ONCOMINE database correlates with decreased survival in prostate cancer compared with PKM2 low (red, *n* = 12) group.

Mount evidence has demonstrated that PKM2 plays a critical role in tumorigenesis (10, 24, 25). To explore the correlation of PKM2 expression with the clinical outcome of prostate cancer patients, immunohistochemical analysis showed that the level of PKM2 expression was elevated in prostate cancer tissues compared with normal tissues (Figure 1A), which is in line with mRNA analysis of PKM2 from the ONCOMINE microarray gene expression dataset^2^ and clinical sample (Figure 1B). Interestingly, PKM2 mRNA level is enriched in tumor metastases (*n* = 35) compared with that in the primary site (*n* = 59) (Figure 1C). Further analysis showed that PKM2 expression in the tumors is correlated with increased Gleason scores (Figure 1D). In addition, the Kaplan–Meier survival analysis showed that overall survival for patients with high PKM2 expression was significantly shorter than those with low PKM2 expression (Figure 1E). These results suggested that PKM2 might be involved in tumor metastasis and predicted poor prognosis in prostate cancer.

### PKM2 Promotes Migration, Invasion, and EMT in Prostate Cancer Cells

**FIGURE 2:**
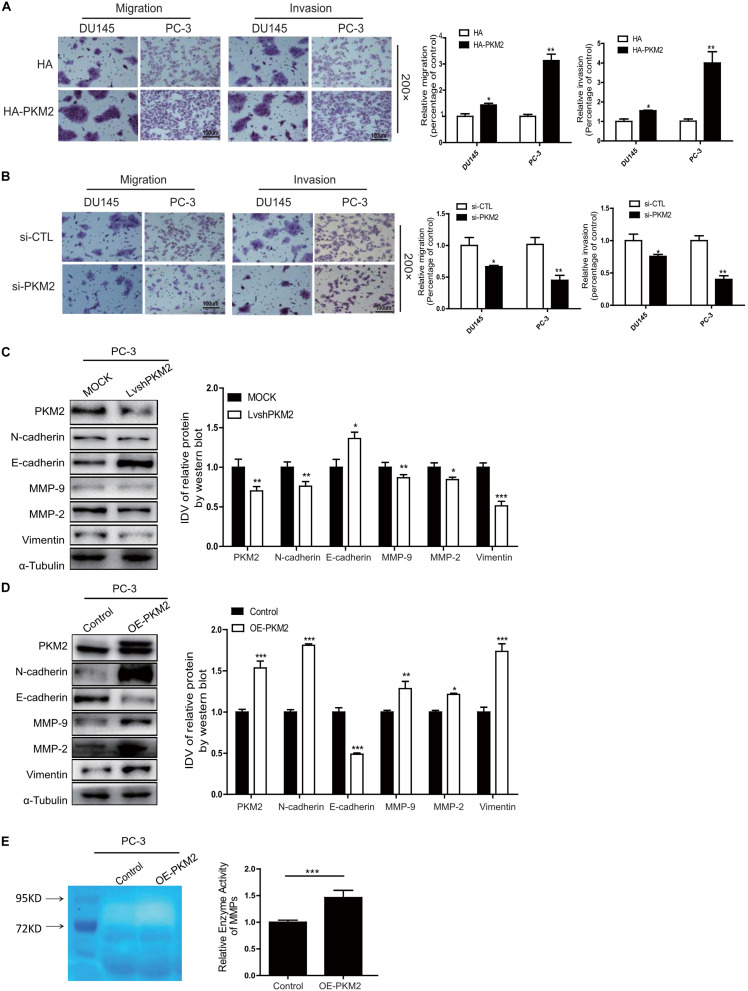
PKM2 promotes EMT, invasion, and migration in prostate cancer cells. DU145 and PC-3 cells were transfected with **(A)** HA-pcDNA3.1 (HA) control or HA-PKM2 plasmid and **(B)** scrambled negative control siRNA (siCTL), specific si-PKM2. After transfection for 48h, cell migration and invasion were assessed by Transwell assays. PC-3 was transduced with **(C)** PKM2 lentivirus (LvshPKM2 or OE-PKM2), or **(D)** control lentivirus (MOCK or CTL) and expression of epithelial–mesenchymal transition (EMT) markers E-cadherin, N-cadherin, vimentin, matrix metalloproteinase protein (MMP)-9, and MMP-2 were detected by Western blot. **(E)** Gelatin zymography was used to detect the expression of MMP-2 and MMP-9 in PKM2-overexpressed stable cells lines. Data were representative of three independent experiments and presented as mean ± SD. Values of p were calculated using paired *t*-test. *p < 0.05, **p < 0.01, ***p < 0.001.

To evaluate the specific effect of PKM2 on migration and invasion as well as EMT in prostate cancer cells, DU145 or PC-3 was transiently transfected with pcDNA3.1-HA (HA) and HA-tagged PKM2 plasmid (HA-PKM2) to determine the migration and invasion using Transwell assay. As shown in Figure 2A, overexpression of PKM2 significantly increased cell migration and invasion compared with the control group in DU145 or PC-3 cells, respectively. In contrast, PKM2 depletion with small interfering RNA (siRNA–PKM2) substantially decreased cell migration and invasion in prostate cancer cells (Figure 2B). Meanwhile, overexpression and knockdown of PKM2 were confirmed at mRNA and protein levels by qRT-PCR or Western blotting (Supplementary Figure 1). We then analyzed the correlation of PKM2 with metastasis gene in clinical prostate cancer, as shown in Supplementary Figures 2A–C. PKM2 is correlated with the expression of metastasis-related gene in prostate cancer. Moreover, PKM2 silencing led to significant downregulation of N-cadherin, vimentin, and MMP2/MMP9, and upregulation of E-cadherin in PC3 cells (Figure 2C). Conversely, the opposite effect on these protein markers were observed in PC3 with PKM2 overexpression compared with control lentivirus (Figure 2D). In addition, the MMP activity was greatly increased after ectopic expression of PKM2 in PC3 cells (Figure 2E). These data indicated that PKM2 contributed to MMP activity and EMT in prostate cancer cells.

### PKM2 Contributes to EMT, Invasion, and Migration in a COX-2-Dependent Manner

**FIGURE 3:**
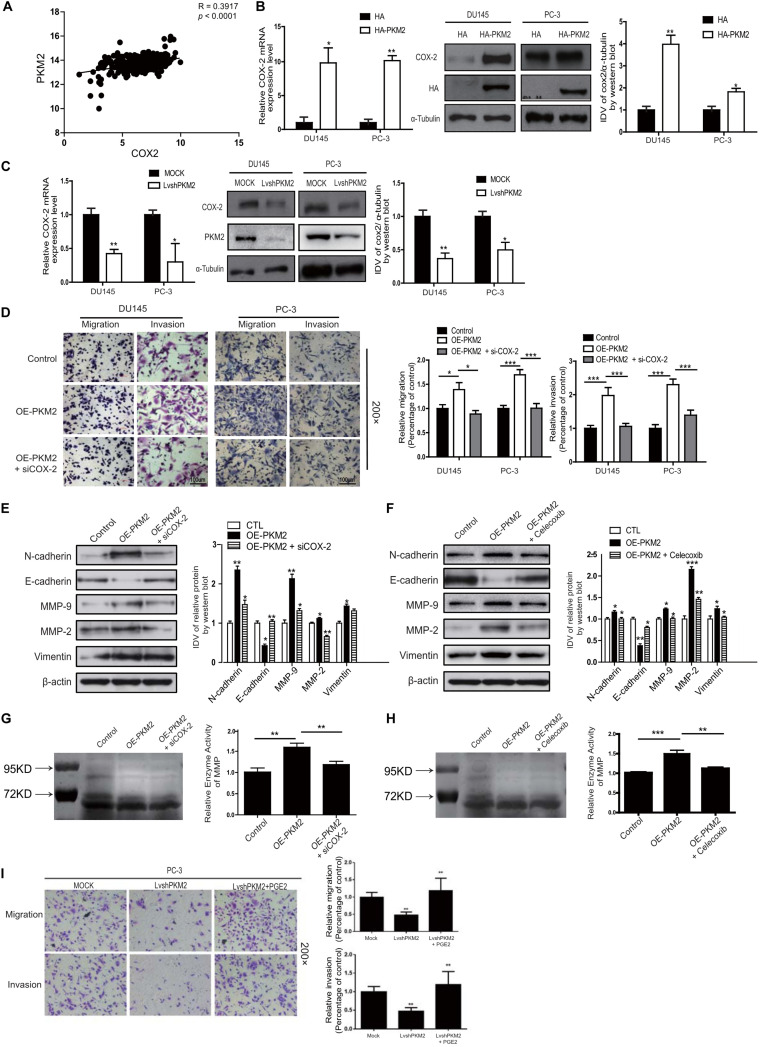
Cyclooxygenase-2 (COX-2) is required for PKM2-induced EMT, invasion, and migration in prostate cancer cells. **(A)** Linear regression and Pearson correlation of mRNA levels between pkm2 and cox-2 in the TCGA database (https://cancergenome.nih.gov/abouttcga/overview). Positive correlation between expression of PKM2 and COX-2 was observed. DU145 and PC-3 cells were transfected with HA and HA-PKM2 plasmid **(B)** or with LvshRNA of PKM2 **(C)** as indicated. After 48 h of transfection, total RNA was extracted and performed to detect COX-2 by real-time PCR, and total protein was collected to detect related COX-2 protein by Western blotting. Data were representative of three independent experiments. Values of p were calculated using paired *t*-test and presented as mean ± SD. *p < 0.05, **p < 0.01, ***p < 0.001. **(D)** DU145 and PC-3 were infected with lentivirus control (Control) or lentivirus OE-PKM2. After 24-h infection, the lentivirus OE-PKM2 group was transiently transfected with siRNA of the control or siRNA of COX-2 (siCOX-2). After another 24-h transfection, cell migration and invasion were assessed by Transwell assays. **(E)** Protein levels of EMT markers E-cadherin, N-cadherin, and vimentin were assayed by Western blotting from PC3 cells infected with lentivirus control (Control) or lentivirus OE-PKM2 with or without transfection of siRNA–COX-2 (siCOX-2). **(F)** Protein levels of EMT markers E-cadherin, N-cadherin, and vimentin were assayed by Western blotting from PC3 cells infected with lentivirus control (Control) or lentivirus OE-PKM2 with or without treatment of COX2 inhibitor (celecoxib). **(G)** The conditioned medium was concentrated and analyzed by gelatinase zymography from PC3 cells infected with lentivirus control (Control) or lentivirus OE-PKM2 with or without transfection of siRNA–COX-2 (siCOX-2). **(H)** The conditioned medium was concentrated and analyzed by gelatinase zymography from PC3 cells infected with lentivirus control (Control) or lentivirus OE-PKM2 with or without treatment of COX-2 inhibitor (celecoxib). **(I)** stable MOCK or shPKM2 (lvshPKM2) PC-3 cells were treated with or without PGE2 (25 nM) for 12 h. Cell migration and invasion assay were determined by Transwell assay. Data represent the mean ± SD of three independent experiments and were analyzed by one-way ANOVA with multiple comparisons, followed by Dunnett’s *post hoc* test for significance versus control. *p < 0.05, **p < 0.01, ***p < 0.001.

Cyclooxygenase 2 (COX-2) was the enzyme implicated in inflammation and a variety of malignancies, especially in invasion and metastasis (26–28). We also analyzed the correlation of PKM2 with COX-2 mRNA expression in different cancers from the TCGA database^1^. The correlation of PKM2 with COX-2 mRNA expression was observed in breast cancer, lung cancer, and colon cancer and is shown in Figure 3A (Supplementary Figure 2D). Moreover, overexpression of PKM2 expression in DU145 and PC-3 cells led to a significant upregulation of COX-2 expression in mRNA (Figure 3B). In contrast, PKM2 depletion substantially decreased the expression of COX-2 mRNA and protein level (Figure 3C). Similar results were obtained in the LNCaP cells (Supplementary Figure 3).

To further explore whether PKM2-mediated EMT and invasion of prostate cancer cells in a COX-2-dependent manner, Transwell assay and Western blotting were employed to confirm the hypothesis. As shown in Figure 3D, COX-2 silencing in DU145 and PC3 cells could reverse the effect of PKM2 in tumor invasion/migration. In line with this, knockdown of COX2 expression in DU145 and PC-3 cells overcame the inhibition of E-cadherin induced by PKM2, while enhancement of N-cadherin, MMP9/2, and vimentin was caused by PKM2 overexpression (Figure 3E). Similar results were obtained in cells with PKM2 overexpression and celecoxib, a potent and highly selective COX-2 inhibitor (29) (Figure 3F and Supplementary Figure 4). In addition, si-COX-2 or celecoxib also remarkably antagonized the gelatinase activity of MMPs induced by overexpression of PKM2 in PC3 cells (Figures 3G,H). Given that prostaglandin E2 (PGE2) is generated from arachidonic acid by the enzyme COX-2, we explored whether exogenous PGE2 antagonizes the inhibitory effect of PKM2 knockdown in PC-3 cells. As shown in Figure 3I, PGE2 treatment restored the attenuated migration and invasion in PKM2 knockdown cells. These results indicated that COX-2 was crucial in mediating the effects of PKM2 on EMT, migration, and invasion in prostate cancer cells.

### PKM2 Regulated COX-2 Expression by Activation of c-Jun

**FIGURE 4:**
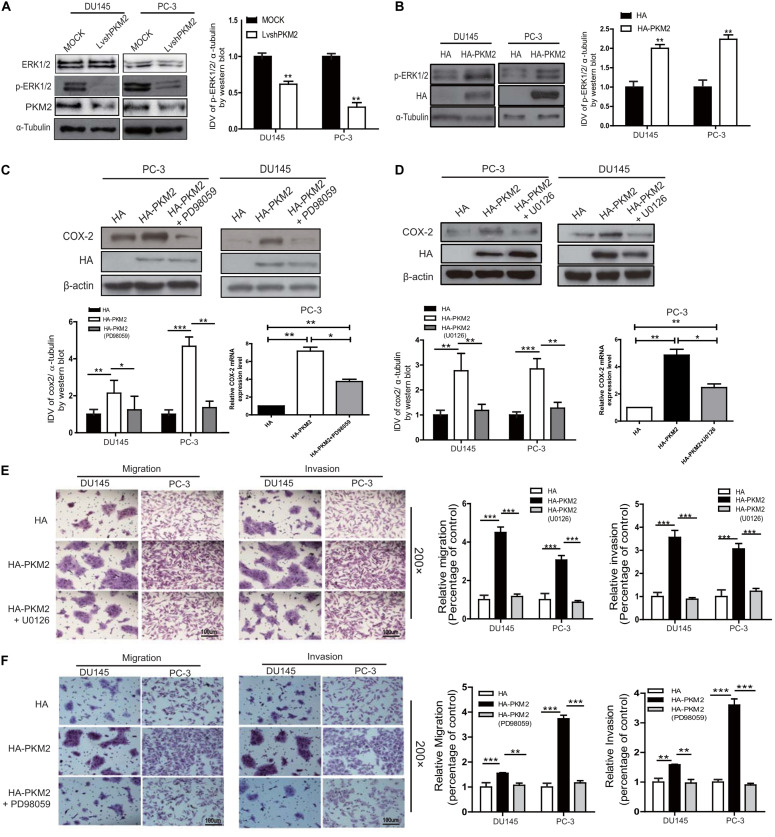
PKM2 enhances COX-2 expression and invasion/migration via extracellular-regulated protein kinase (ERK)1/2 signaling in prostate cancer cells. DU145 and PC-3 cells were transfected with HA-tagged PKM2 or lentivirus PKM2. **(A,B)** Immunoblotting of DU145 and PC-3 cell lysates for phosphorylation levels of ERK1/2. Data were representative of three independent experiments. DU145 and PC-3 cells were transfected with HA or HA-tagged PKM2 followed by stimulation of ERK1/2 inhibitor U0126 (1.5 μM) **(D,E)** and PD98059 (50 μM) **(C,F)**, respectively. **(C,D)** Protein and mRNA levels of COX-2 were measured by Western blotting as well as q-RT-PCR. **(E,F)** Cell invasion and migration were assessed by Transwell assays. Values of p were calculated using paired *t*-test and presented as mean ± SD. *p < 0.05, **p < 0.01, ***p < 0.001.

It has been shown that ERK1/2 is involved in regulating COX-2 expression in various tumor cells (30, 31). To further explore whether ERK1/2 was involved in PKM2-mediated COX-2 expression, as shown in Figures 4A,B, overexpression of PKM2 in DU145 and PC-3 cells led to phosphorylation of ERK1/2, while depletion of PKM2 expression inhibited phosphorylation of ERK1/2. Similar results were obtained in LNCaP cells (Supplementary Figures 5A,B).

To determine whether the upregulation of COX-2 was due to PKM2-mediated activation of ERK1/2 signaling, DU145 and PC-3 cells were transfected with PKM2 plasmid and then treated with the MEK inhibitor PD98059 or U0126. As shown in Figures 4C,D, the expression of COX-2 mRNA and protein level was markedly blocked by PD98059 or U0126 in PC3 and DU145 cells triggered by overexpression of PKM2. Similarly, the Transwell assay also showed that enhanced cell migration and invasion induced by PKM2 overexpression were also significantly reduced by PD98059 or U0126 in PC3 and DU145 cells (Figures 4E,F). Collectively, these data indicated that PKM2 regulated prostate cancer cell migration and invasion through the ERK1/2-COX-2 signaling axis.

### PKM2 Interacts With ERK1/2 and Is Required for Binding of c-Jun to the Promoter of cox-2 Gene

**FIGURE 5:**
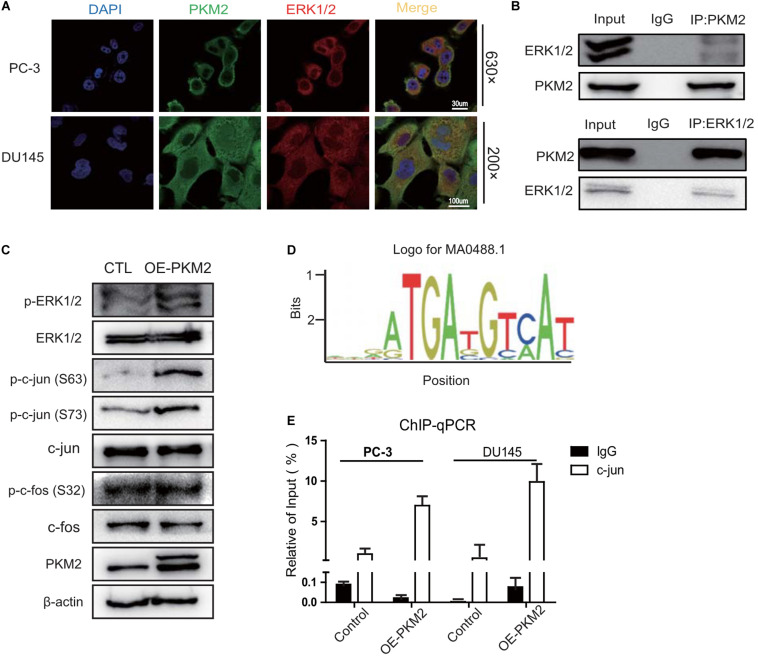
PKM2 interacts with ERK1/2 and promotes the binding of c-Jun to the promoter of COX-2 gene. **(A)** Colocalization of PKM2 and ERK1/2 by immunofluorescence in DU145 and PC-3 cells. **(B)** PC-3 cells were grown to 70–80% confluence; whole cell lysates were immunoprecipitated (IP) with antibodies against endogenous PKM2 or ERK1/2 detected by immunoblotting. **(C)** Immunoblotting assay of ERK1/2, p-ERK1/2, c-Jun, p-c-Jun, c-Fos, p-c-Fos in the Control (CTL), or PKM2-overexpressing PC-3 cells. **(D)** A putative consensus sequence GGAATGATG of c-Jun binding in the promoter of COX-2 by the JASPAR database. **(E)** c-Jun binding to the promoter of COX-2 was analyzed by CHIP assay in DU145 and PC-3 cells infected with lentivirus control or overexpressed PKM2.

Given that PKM2 upregulated the expression of COX-2 through ERK1/2 signaling, we evaluated the possible interaction of PKM2 with ERK1/2 in prostate cancer cells. Immunofluorescence assay showed that endogenous PKM2 is colocalized with ERK1/2 in both nucleus and cytoplasm of PC-3 or DU145 cells (Figure 5A). Also, co-immunoprecipitation demonstrated that endogenous PKM2 interacted with ERK1/2 in PC-3 cells (Figure 5B). Moreover, we transfected different PKM2 mutant plasmids and detected the phosphorylation of ERK1/2. As shown in Supplementary Figure 6A, full length (FL) and the N terminal of PKM2 (NABD) increased the phosphorylation of ERK1/2. In line with this effect, the full length and NABD mutant interacted with ERK1/2 (Supplementary Figure 6B).

We further determined whether PKM2 promotes phosphorylation of c-Jun or c-Fos downstream of ERK1/2, which heterodimerize to form the AP1 transcriptional factor for binding to the promoter of target genes (32). As shown in Figure 5C, overexpression of PKM2 increased the phosphorylation of c-Jun at ser63 and ser73, but not the phosphorylation of c-Fos at ser32 compared with the control in PC-3 cells. To further investigate whether phosphorylation of c-Jun induced by PKM2 promotes the binding of c-Jun to the promoter of *cox-2* gene, a putative consensus sequence GGAATGATG of c-Jun-binding element (SBE) in the promoter of *cox-2* gene was speculated by the JASPAR database (Figure 5D). CHIP assay demonstrated that the binding of c-Jun to the promoter of *cox-2* gene was markedly increased by overexpression of PKM2 in PC-3 and DU145 cells (Figure 5E). These data indicated that PKM2 regulated the expression of COX-2 via ERK1/2 signaling, leading to the phosphorylation of c-Jun and its binding to the promoter of *cox-2* gene.

### Inhibition of COX-2 Attenuated PKM2-Mediated Metastasis *in vivo*

**FIGURE 6:**
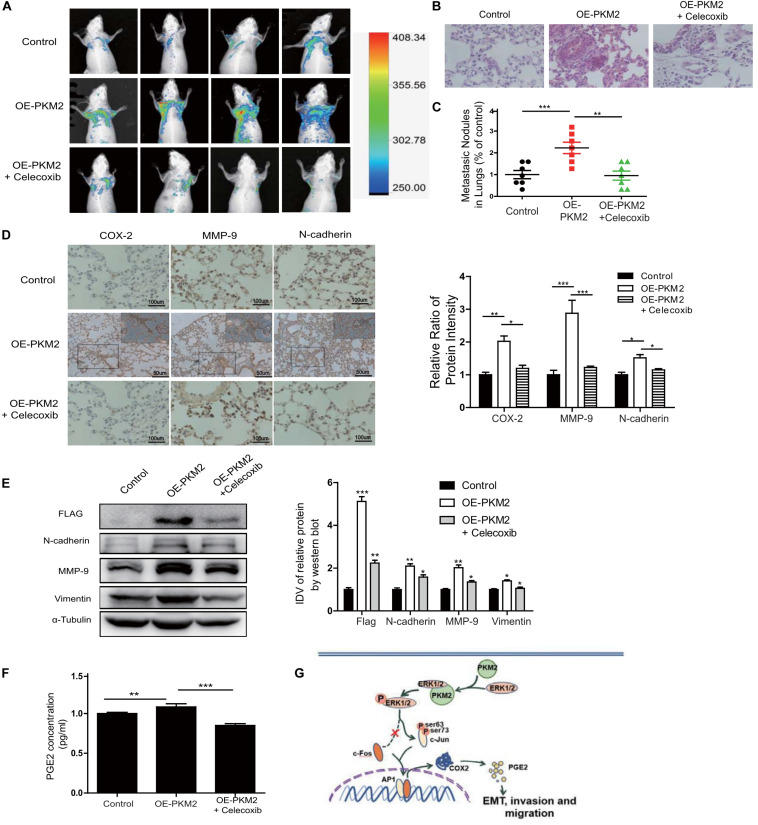
Overexpression of PKM2 accelerates tumor metastasis, COX-2 expression, and EMT *in vivo*. **(A)** Representative mouse from each treatment group demonstrating fluorescence signal at day 21 after tumor cell inoculation. Treated with celecoxib, mice showed less fluorescence of GFP in the lungs than in OE-PKM2 mice. **(B)** HE staining of the lungs of mouse models was performed. **(C)** Metastatic nodules in the lungs were counted under microscope and quantitatively analyzed. **(D)** IHC staining detected COX-2, MMP9, and N-cadherin expression in various groups of lung metastatic nodules in a mouse inoculated with PKM2-transduced PC-3 cell. Right: the bar graph shows the relative ratio of staining intensity of COX-2, MMP9, and N-cadherin diaminobenzidine (DAB) in each group. **(E)** EMT marker, MMP9, and Flag tag protein were detected by Western blotting in lung tissue. **(F)** Measurements of the levels of PGE2 in the serum of each group. **(G)** Schematic illustration for PKM2 promotes prostate cancer metastasis through regulating ERK1/2-COX-2 signaling. In prostate cancer, elevated expression of PKM2 interacts with ERK1/2 and contributes to the phosphorylation of ERK1/2, leading to the phosphorylation of subsequent downstream c-Jun and binding to the promoter of *cox-2* gene. Upregulation of PGE2 by COX-2 promotes EMT, invasion, and migration of tumor cells. Data represent the mean ± SEM of three independent experiments and were analyzed by one-way ANOVA with multiple comparisons, followed by Dunnett’s *post hoc* test for significance versus control and presented as mean ± SD. *p < 0.05, **p < 0.01, ***p < 0.001.

To determine whether PKM2-mediated expression and activity of COX-2 play a critical role in tumor metastasis *in vivo*, we ectopically expressed GFP and PKM2 (OE-PKM2) using lentiviruses in PC3 cells and then established stable expression clones (*n* = 6 per group). Control or OE-PKM2 of PC-3/GFP-expressing cells were injected into the tail vein of male athymic nude mice, followed by treatment with celecoxib (12.5 mg/kg/day) or vehicle for 21 days. The result showed that there were no significant differences in the body weight among the three groups (Supplementary Figure 7). *In vivo* live image analysis showed that overexpression of PKM2 increased the fluorescence of GFP signal manifested by representative fluorescence images, whereas celecoxib treatment antagonized the enhanced effect of PKM2 on the lung metastatic lesion of PC3 cells in mice (Figure 6A). Moreover, H&E staining and quantitative analysis of the metastatic nodules in lungs showed that celecoxib treatment significantly reduced the number of metastatic nodules in the lungs of prostate cancer patients induced by of PKM2 overexpression (Figures 6B,C), while N-cadherin, vimentin, and MMP9 were also markedly inhibited in response to celecoxib treatment (Figures 6D,E). In addition, celecoxib significantly antagonized the level of COX-2 and its product PGE2 in the serum of mice injected with PKM2-overexpressed PC3 cells (Figure 6F). Taken together, these data demonstrated that PKM2 promoted tumor metastasis through upregulation of COX-2 and MMP2 to promote prostate cancer metastasis *in vivo*.

## Discussion

Most cancer cells with high levels of PKM2 expression promoted aerobic glycolysis, resulting in selective advantage over cancers with low levels of PKM2 (33–35). However, the mechanism through which PKM2 contributes to EMT and tumor metastasis remains largely unknown. Here, as shown in Figure 6G, for the first time, we reported that a novel PKM2–COX-2 axis promoted EMT and metastasis of prostate cancer *in vitro* and *in vivo*. PKM2 interacted with ERK1/2, leading to phosphorylation of c-Jun (ser63) and binding to the promoter of the *cox-2* gene, thereby resulting in significant tumor migration and invasion. Importantly, inhibition of COX-2 greatly reduced tumor metastasis caused by PKM2 *in vivo*. In summary, our data suggested that targeting the PKM2 signaling would be a potential therapy in metastatic prostate cancer.

The tetrameric form of PKM2 catalyzed the conversion of phosphoenolpyruvate (PEP) to pyruvate by transferring a phosphate from PEP to ADP (36), whereas the dimeric form is nearly inactive. Meanwhile, dimeric PKM2 can undergo translocation to the nucleus and functions as a protein kinase and transcriptional coactivator, and promotes proliferation and tumorigenesis (37). Until this date, PKM2 has been reported to be phosphorylated at the Ser37 by ERK, leading to nuclear localization, in response to the Warburg effect and tumorigenesis (38). Interestingly, we further showed that PKM2 suppression significantly reduced phosphorylation of ERK1/2 level, whereas PKM2 overexpression increased phosphorylation of ERK1/2, indicating the positive regulation loop between PKM2 and ERK1/2 in prostate cancer. Further work is required to address this. What is more, further analysis showed that only NABD, not the ABD and CD domains of PKM2, interacts with ERK1/2, implying the critical role of the NABD domain of PKM2 in tumor metastasis. Nevertheless, in our next work, we would demonstrate the effect of the complex on either PKM2 or ERK1/2 modification, including phosphorylation, methylation, and acetylation.

Epithelial–mesenchymal transition is an essential event during cancer metastasis. Although overexpression of PKM2 has been associated with increased EMT and tumor migration/invasion (39, 40), the exact role of PKM2 in prostate cancer metastasis and the mechanism by which it exerts its prometastatic function in prostate cancer cells is yet to be determined. Our study showed that knockdown PKM2 leads to inhibition of motility in PC-3 cells and the EMT marker. Thus, it is reasonable to deduce that PKM2 regulates EMT as well as the motility of PC-3. It has been shown that PKM2 interacts with the transcriptional factor TGF-β-induced factor homeobox 2, resulting in increased E-cadherin expression (40). In addition, PKM2 functions as an interacting partner of EGFR in the nucleus of irradiation-resistant cells to regulate the transcription of stemness-related genes and promote the stem-like phenotype, thus promoting invasion and metastasis (41). These studies further support our findings that PKM2 promotes tumor metastasis through non-glycolytic pathways, specifically the PKM2–ERK1/2–COX-2 pathway in prostate cancer cells.

Our data suggested that PKM2 played a significant role in promoting migration potential and invasiveness of prostate cancer cells, in part, through ERK1/2 pathway activation becausePKM2 interacted with ERK1/2 and colocalized with ERK1/2 in prostate cancer cells. c-Jun, a substrate of EKR1/2, forms the AP-1 early response transcription factor and plays a crucial role in the development and progression of cancer (42–45). Previous studies have shown that cancer-relevant genes, including COX-2, are regulated by c-Jun (46). Importantly, in the present study, we identified that PKM2-dependent ERK1/2 phosphorylation was essential for c-Jun-mediated COX-2 expression. The identified PKM2 is potentially required for c-Jun direct binding to the promoter of the *cox-2* gene. In addition, previous experiments have shown that EMT is associated with COX-2 (47). Rong et al. (48) demonstrated that PKM2 induced EMT by regulating STAT3. Cheng et al. (49) further reported the elevated expression of PKM2/STAT3/Snail in TGF-β1-induced EMT. In the present study, PKM2 promoted the phosphorylation of ERK1/2 to increase the transcription of COX-2 that leads to EMT and metastasis of prostate cancer. Furthermore, this was abolished upon PKM2 knockdown.

Considering that celecoxib attenuates metastasis, we assess the effect of celecoxib on invasion and migration in PKM2 knockdown cells. Interestingly, the PKM2 expression was not effected in cells treated with celecoxib or CHX (Supplementary Figure 8A). Additional targeting of COX-2 did not attenuate migration and invasion if PKM2 is absent (Supplementary Figure 8B). Thus, we suggested that PKM2–COX-2 is the major axis in the metastasis of prostate cancer.

In summary, we have demonstrated that a novel pathway through which PKM2 induced COX-2 expression is by activation of the ERK1/2–c-Jun axis. PKM2 interacted with ERK1/2 and regulated ERK1/2 activity, leading to the phosphorylation of c-Jun, not c-Fos, initiating transactivation of COX-2, and promoting cell invasion/EMT, while inhibition of COX-2 reversed the promotion of PKM2 on tumor invasion/EMT *in vivo* and *in vitro*. However, further studies are required to determine whether additional c-Jun targets are also controlled by the PKM2-mediated mechanism. Nevertheless, the current study provides novel insights into the non-glycolytic role of PKM2 in cancer cells.

## Data Availability Statement

All datasets presented in this study are included in the article/Supplementary Material.

## Ethics Statement

The animal study was reviewed and approved by The Institutional Animal Care and Use Committee of Southern Medical University.

## Author Contributions

FZ and FD participated in the research design. WG, ZZ, GL, WX, XL, RG, HC, ZX, LC, and JQ conducted the experiments. WG, ZZ, WX, SX, and FZ analyzed the data. WG, ZZ, and FD wrote or contributed to the writing of the manuscript. All authors contributed to the article and approved the submitted version.

## Conflict of Interest

The authors declare that the research was conducted in the absence of any commercial or financial relationships that could be construed as a potential conflict of interest.

## Footnotes

1
https://cancergenome.nih.gov/abouttcga/overview

2
www.oncomine.org

## Abbreviations

PKM2: pyruvate kinase M2
COX-2: cyclooxygenase-2
ERK: extracellular-regulated protein kinase
EMT: epithelial–mesenchymal transition
PCa: prostate cancer
ADT: androgen deprivation therapy
ECM: extracellular matrix
MMP: matrix metalloproteinase proteins
MAPK: mitogen-activated protein kinase
AP-1: activator protein 1.
