# Corrigendum: Pyruvate kinase M2 promotes prostate cancer metastasis through regulating ERK1/2-COX-2 signaling

**DOI:** 10.3389/fonc.2024.1323020

**Published:** 2024-02-12

**Authors:** Wenjing Guo, Zhishuai Zhang, Guihuan Li, Xiaoju Lai, Ruonan Gu, Wanfu Xu, Hua Chen, Zhe Xing, Liping Chen, Jiabi Qian, Shiyuan Xu, Fangyin Zeng, Fan Deng

**Affiliations:** ^1^ Department of Cell Biology, School of Basic Medical Sciences, Southern Medical University, Guangzhou, China; ^2^ Department of Anesthesiology, Zhujiang Hospital, Southern Medical University, Guangzhou, China; ^3^ Department of Clinical Laboratory, the Fifth Affiliated Hospital, Southern Medical University, Guangzhou, China

**Keywords:** pyruvate kinase M2, ERK1/2, cyclooxygenase 2, tumor metastasis, prostate cancer

**Error in Figure**

In the published article, there was an error in **Figure 3F**, **Figure 6D** as published. **Figure 3F** band of MMP-9 and COX-2 IHC image of Control group in **Figure 6D** were mistakenly displayed, respectively. The corrected **Figure 3** and **Figure 6** and their captions appear below.

**Figure 3:**
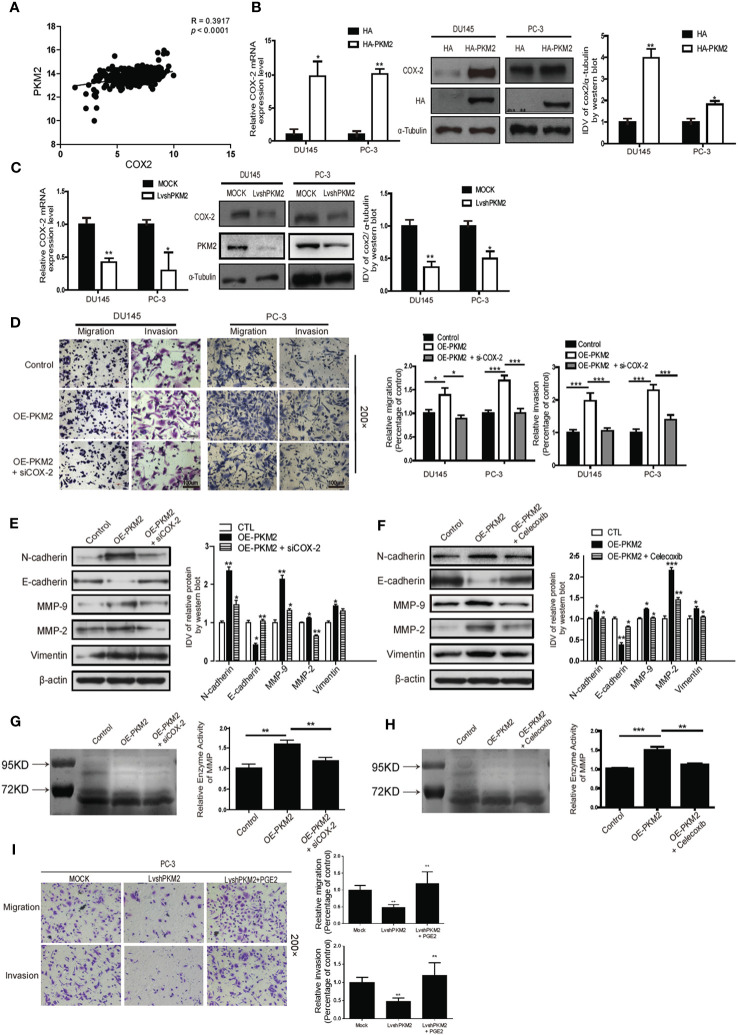
Cyclooxygenase-2 (COX-2) is required for PKM2-induced EMT, invasion, and migration in prostate cancer cells. **(A)** Linear regression and Pearson correlation of mRNA levels between pkm2 and cox-2 in the TCGA database (https://cancergenome.nih.gov/abouttcga/overview). Positive correlation between expression of PKM2 and COX-2 was observed. DU145 and PC-3 cells were transfected with HA and HA-PKM2 plasmid **(B)** or with LvshRNA of PKM2 **(C)** as indicated. After 48 h of transfection, total RNA was extracted and performed to detect COX-2 by real-time PCR, and total protein was collected to detect related COX-2 protein by Western blotting. Data were representative of three independent experiments. Values of p were calculated using paired t-test and presented as mean ± SD. *p < 0.05, **p < 0.01, ***p < 0.001. **(D)** DU145 and PC-3 were infected with lentivirus control (Control) or lentivirus OE-PKM2. After 24-h infection, the lentivirus OE-PKM2 group was transiently transfected with siRNA of the control or siRNA of COX-2 (siCOX-2). After another 24-h transfection, cell migration and invasion were assessed by Transwell assays. **(E)** Protein levels of EMT markers E-cadherin, N-cadherin, and vimentin were assayed by Western blotting from PC3 cells infected with lentivirus control (Control) or lentivirus OE-PKM2 with or without transfection of siRNA–COX-2 (siCOX-2). **(F)** Protein levels of EMT markers E-cadherin, N-cadherin, and vimentin were assayed by Western blotting from PC3 cells infected with lentivirus control (Control) or lentivirus OE-PKM2 with or without treatment of COX2 inhibitor (celecoxib). **(G)** The conditioned medium was concentrated and analyzed by gelatinase zymography from PC3 cells infected with lentivirus control (Control) or lentivirus OE-PKM2 with or without transfection of siRNA–COX-2 (siCOX-2). **(H)** The conditioned medium was concentrated and analyzed by gelatinase zymography from PC3 cells infected with lentivirus control (Control) or lentivirus OE-PKM2 with or without treatment of COX-2 inhibitor (celecoxib). **(I)** stable MOCK or shPKM2 (lvshPKM2) PC-3 cells were treated with or without PGE2 (25 nM) for 12 h. Cell migration and invasion assay were determined by Transwell assay. Data represent the mean ± SD of three independent experiments and were analyzed by one-way ANOVA with multiple comparisons, followed by Dunnett’s *post hoc* test for significance versus control. *p < 0.05, **p < 0.01, ***p < 0.001.

**Figure 6:**
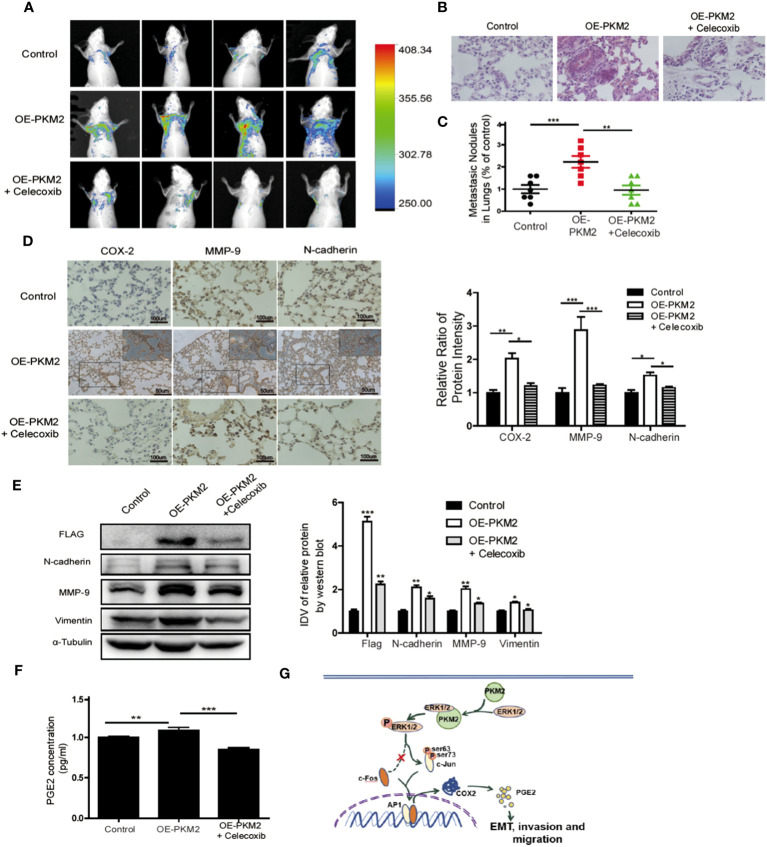
Overexpression of PKM2 accelerates tumor metastasis, COX-2 expression, and EMT *in vivo*. **(A)** Representative mouse from each treatment group demonstrating fluorescence signal at day 21 after tumor cell inoculation. Treated with celecoxib, mice showed less fluorescence of GFP in the lungs than in OE-PKM2 mice. **(B)** HE staining of the lungs of mouse models was performed. **(C)** Metastatic nodules in the lungs were counted under microscope and quantitatively analyzed. **(D)** IHC staining detected COX-2, MMP9, and N-cadherin expression in various groups of lung metastatic nodules in a mouse inoculated with PKM2-transduced PC-3 cell. Right: the bar graph shows the relative ratio of staining intensity of COX-2, MMP9, and N-cadherin diaminobenzidine **(DAB)** in each group. **(E)** EMT marker, MMP9, and Flag tag protein were detected by Western blotting in lung tissue. **(F)** Measurements of the levels of PGE2 in the serum of each group. **(G)** Schematic illustration for PKM2 promotes prostate cancer metastasis through regulating ERK1/2-COX-2 signaling. In prostate cancer, elevated expression of PKM2 interacts with ERK1/2 and contributes to the phosphorylation of ERK1/2, leading to the phosphorylation of subsequent downstream c-Jun and binding to the promoter of cox-2 gene. Upregulation of PGE2 by COX-2 promotes EMT, invasion, and migration of tumor cells. Data represent the mean ± SEM of three independent experiments and were analyzed by one-way ANOVA with multiple comparisons, followed by Dunnett’s *post hoc* test for significance versus control and presented as mean ± SD. *p < 0.05, **p < 0.01, ***p < 0.001.

The authors apologize for this error and state that this does not change the scientific conclusions of the article in any way. The original article has been updated.

