# Supplementary material for: Pyruvate Kinase M2 Promotes Prostate Cancer Metastasis Through Regulating ERK1/2-COX-2 Signaling
Source: Front Oncol. 2020 Sep 29;10:544288. doi: 10.3389/fonc.2020.544288 (PMC7550821; doi:10.3389/fonc.2020.544288)
Supplement: Supplementary file 1 [file Data_Sheet_1.docx]

Supplementary Material

# Supplementary Figures

**
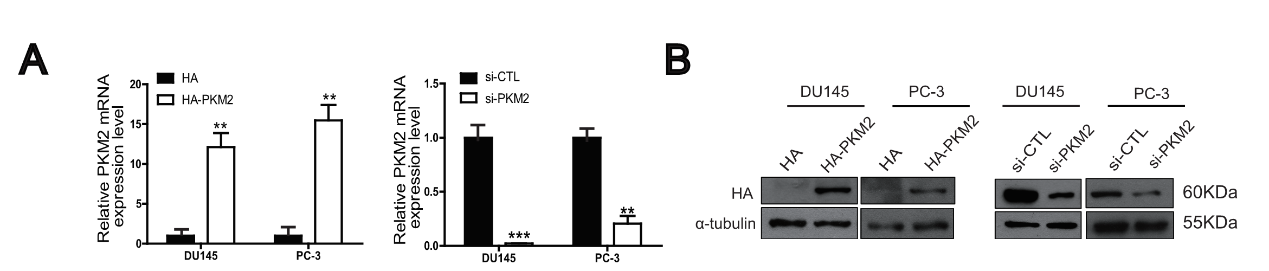
**

**Supplementary Figure 1.** **qRT-PCR and Western blotting analysis of PKM2 expression in DU145 and PC-3.** DU145 and PC3 were transfected with control plasmid (HA-pcDNA-3.1) or HA-PKM2 plasmid and siRNA. The expression levels of pkm2 were detected by qRT-PCR **(A)**. Cell lysates were prepared and subjected to western blotting with anti-HA, anti-PKM2, anti-αtubulin antibodies **(B)**.


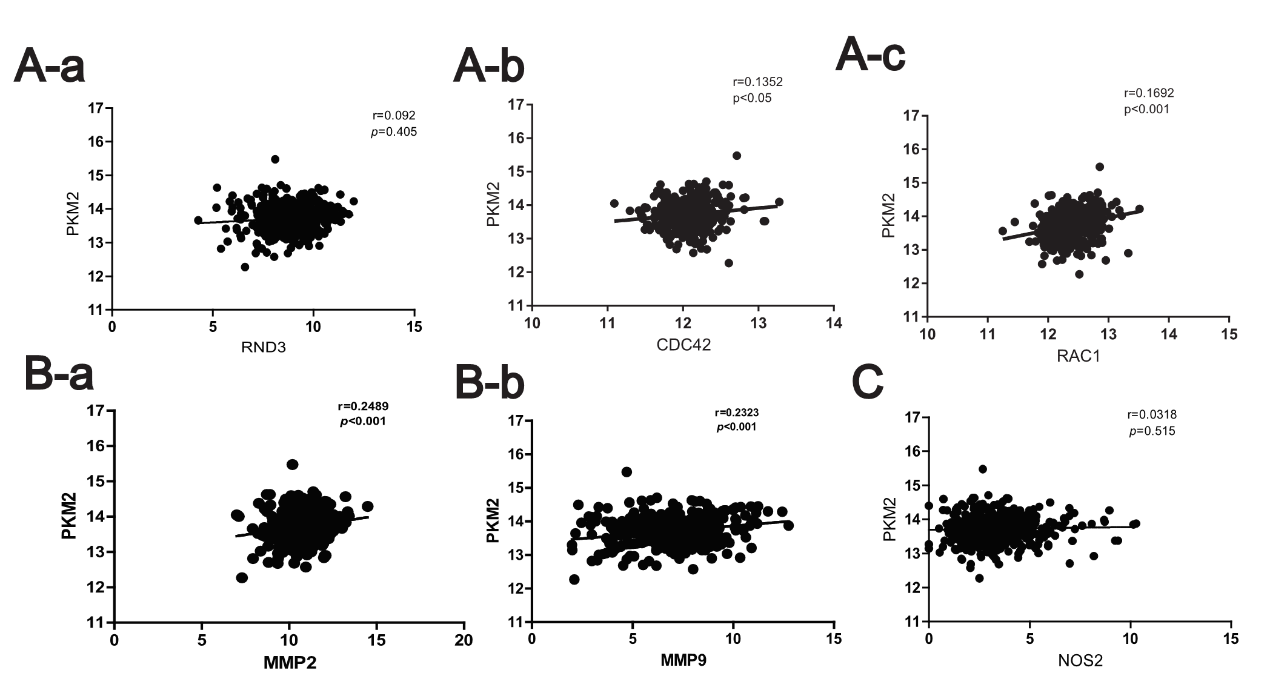


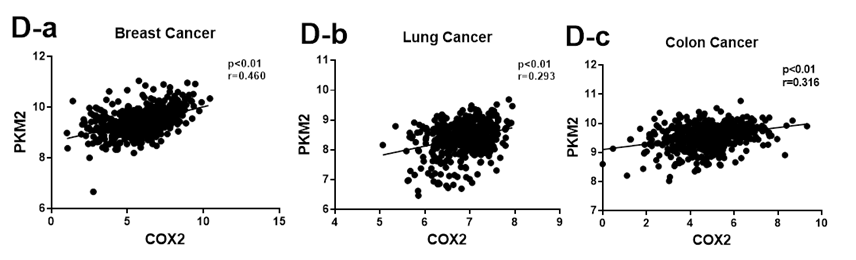


**Supplementary Figure 2** **Correlation of PKM2 with Rho proteins, MMPs, NOS2 level and correlation between PKM2 and COX-2 in other types of cancers in the TCGA prostate cancer database.** Co-expression data between PKM2 and RND3 **(A-a)**, CDC42 **(A-b)**, RAC1 **(A-c)**, MMP2 **(B-a)**, MMP9 **(B-b)** and NOS2 **(C)**, respectively, in the prostate cancer TCGA database. **correlation between PKM2 and COX-2 in breast Cancer (D-a), lung cancer (D-b), colon cancer (D-c).**


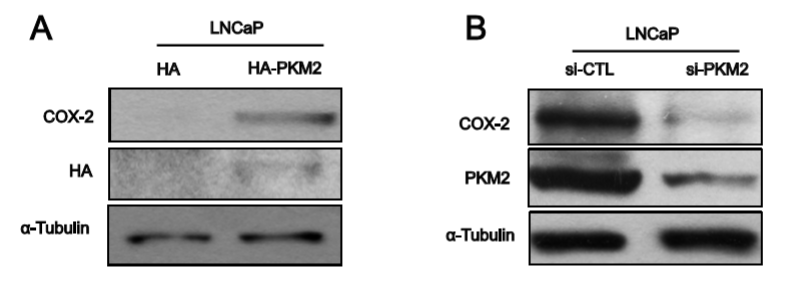


**Supplementary Figure 3 Western blot analysis in LNCaP cells overexpressed with PKM2 or PKM2 silencing.**  LNCaP were transfected with **(A)** HA-tagged PKM2 or **(B)** si-PKM2. Immunoblotting cell lysates for levels of COX-2 and PKM2 expression.


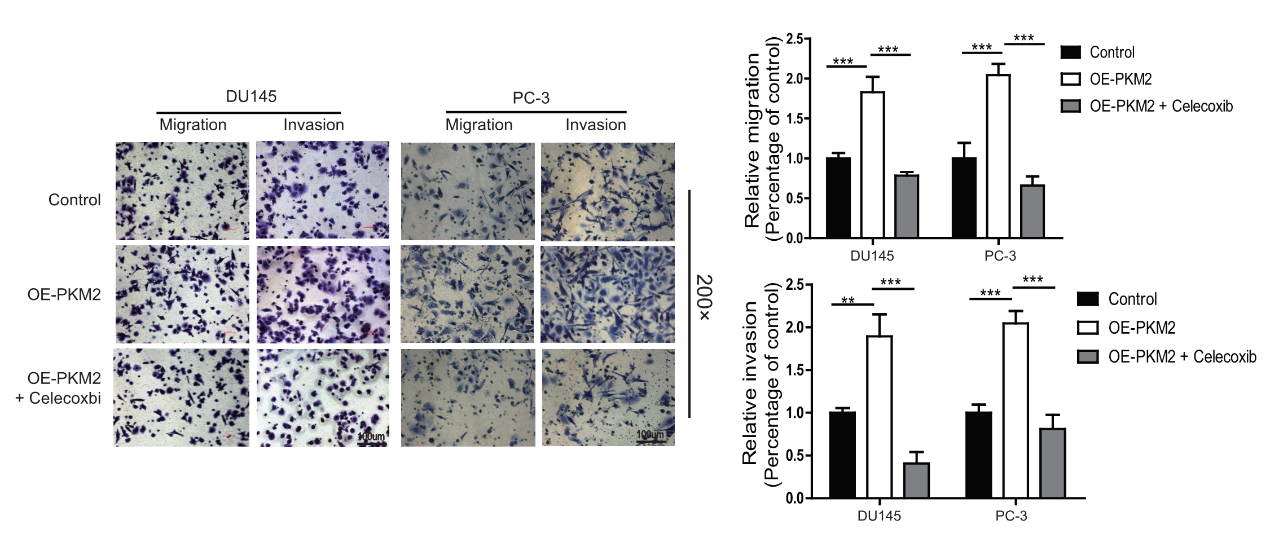


**Supplementary Figure 4 COX-2 inhibitor antagonizes the migration and invasion of prostate cancer cells triggered by overexpression of PKM2.** PKM2-overexpression stable PC-3 and DU145 cell lines (OE-PKM2) or control cell lines (control) were treated by celecoxib and cell migration and invasion was assessed by Transwell assays.


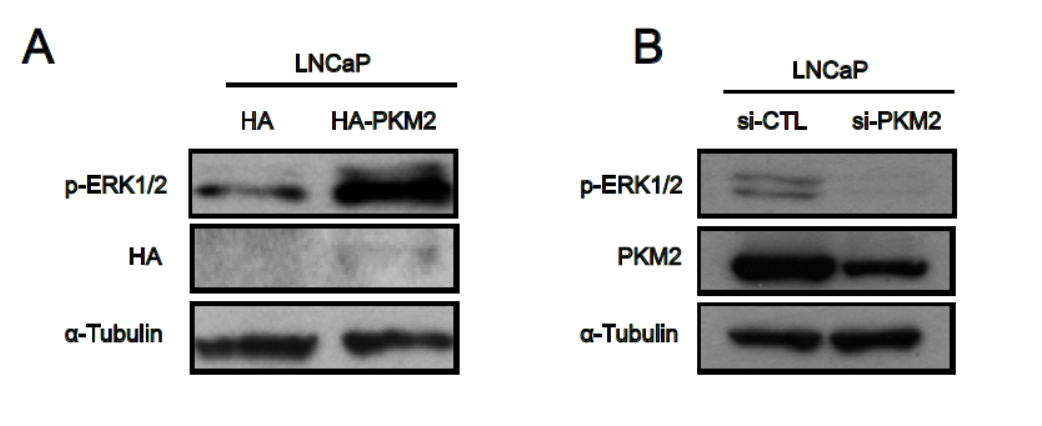


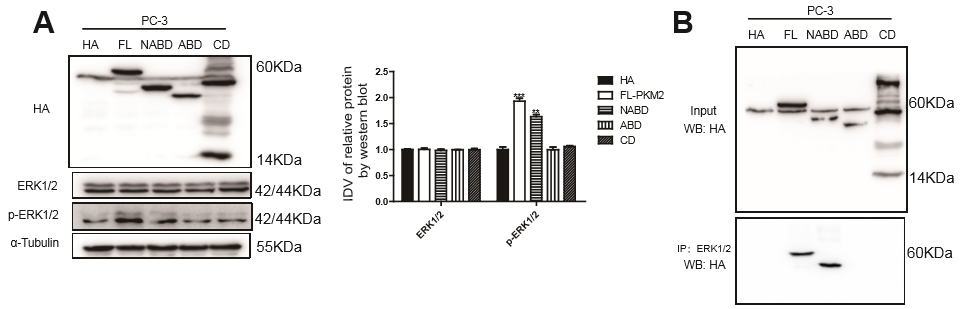
**Supplementary Figure 5 PKM2 upregulates ERK1/2 phosphorylation in LNCaP cells.** Western blot analysis in LNCaP cells. LNCap were transfected with **(A)** HA-tagged PKM2 or **(B)** si-PKM2. Immunoblotting cell lysates for phosphorylation levels of ERK1/2.

**Supplementary Figure 6 NABD domain of PKM2 mediates ERK1/2 phosphorylation in PC3 cells.**  Western blot analysis of ERK1/2 and p-ERK1/2 in PC-3 cells. PC-3 were transfected with HA-pcDNA-3.1(HA), HA-tagged PKM2 (FL) and HA-tagged PKM2 mutant plasmid (NABD, ABD and CD). Immunoblotting cell lysates for ERK1/2 and p-ERK1/2 levels (A). **NABD domain of PKM2 interacted with ERK1/2 in PC3 cells.**  The whole cell lysates were immunoprecipitated (IP) with antibody ERK1/2 and detected HA expression by immunoblotting (B).


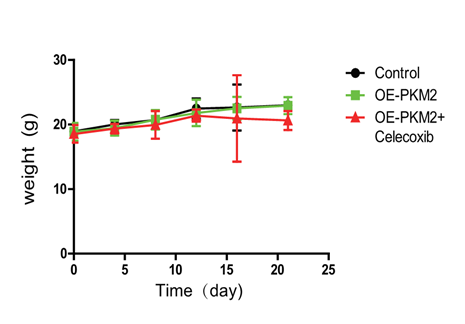


**Supplementary Figure 7 Mice weights are measured in nude mice.** Tail vain injection by using PKM2 overexpressed PC-3 cells that were stably transfected with vector control, flowing by celecoxib in nude mice. The mice were grouped randomly. Mice were measured the weight 5 days.


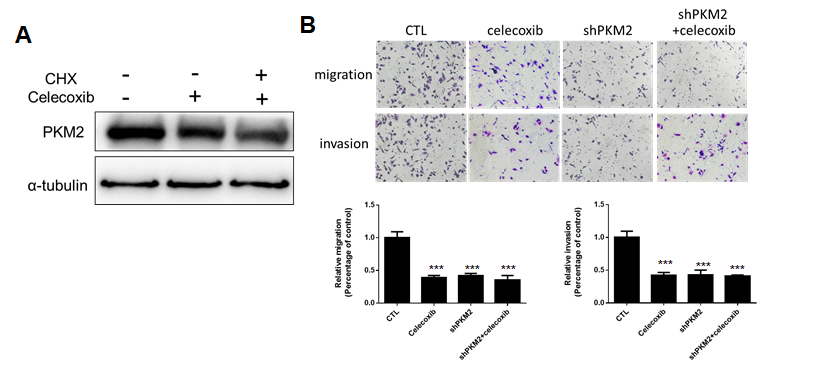


**Supplementary Figure 8 PKM2 expression and cell migration/invasion were inhibited after celecoxib treatment in PC-3**. Celecoxib or combination with CHX treatment decreases PKM2 expression (A). PKM2 knockdown cell were treated with celecoxib and cell migration and invasion were detected by transwell assay (B).
